# Detection of Alveolar Fibrocytes in Idiopathic Pulmonary Fibrosis and Systemic Sclerosis

**DOI:** 10.1371/journal.pone.0053736

**Published:** 2013-01-16

**Authors:** Raphael Borie, Christophe Quesnel, Sophie Phin, Marie-Pierre Debray, Joelle Marchal-Somme, Kiet Tiev, Marcel Bonay, Aurélie Fabre, Paul Soler, Monique Dehoux, Bruno Crestani

**Affiliations:** 1 Assistance Publique-Hopitaux de Paris, Service de Pneumologie A, Centre de Compétences Maladies Rares Pulmonaires, Hôpital Bichat, Paris, France; 2 INSERM, Unité 700, Faculté Bichat, Université Paris 7, Paris, France; 3 Assistance Publique-Hopitaux de Paris, Service d’Anesthésie et de Réanimation Chirurgicale, Hôpital Tenon, Paris, France; 4 Assistance Publique-Hopitaux de Paris, Service de Radiologie, Hôpital Bichat, Paris, France; 5 Assistance Publique-Hopitaux de Paris, Service de Médecine Interne, Hôpital Saint Antoine, Paris, France; 6 Assistance Publique-Hopitaux de Paris, Service d'Explorations Fonctionnelles, Hôpital Saint Antoine, Paris, France; 7 Université Paris Diderot Paris 7, PRES Sorbonne Paris Cité, Paris, France; 8 Assistance Publique-Hopitaux de Paris, Service d'Anatomopathologie, Hôpital Bichat, Paris, France; 9 Assistance Publique-Hopitaux de Paris, Service de Biochimie, Hôpital Bichat, Paris, France; Helmholtz Zentrum München/Ludwig-Maximilians-University Munich, Germany

## Abstract

**Background:**

Fibrocytes are circulating precursors for fibroblasts. Blood fibrocytes are increased in patients with idiopathic pulmonary fibrosis (IPF). The aim of this study was to determine whether alveolar fibrocytes are detected in broncho-alveolar lavage (BAL), to identify their prognostic value, and their potential association with culture of fibroblasts from BAL.

**Methods:**

We quantified fibrocytes in BAL from 26 patients with IPF, 9 patients with Systemic Sclerosis(SSc)-interstitial lung disease (ILD), and 11 controls. BAL cells were cultured to isolate alveolar fibroblasts.

**Results:**

Fibrocytes were detected in BAL in 14/26 IPF (54%) and 5/9 SSc patients (55%), and never in controls. Fibrocytes were in median 2.5% [0.4–19.7] and 3.0% [2.7–3.7] of BAL cells in IPF and SSc-ILD patients respectively. In IPF patients, the number of alveolar fibrocytes was correlated with the number of alveolar macrophages and was associated with a less severe disease but not with a better outcome. Fibroblasts were cultured from BAL in 12/26 IPF (46%), 5/9 SSc-ILD (65%) and never in controls. The detection of BAL fibrocytes did not predict a positive culture of fibroblasts.

**Conclusion:**

Fibrocytes were detected in BAL fluid in about half of the patients with IPF and SSc-ILD. Their number was associated with less severe disease in IPF patients and did not associate with the capacity to grow fibroblasts from BAL fluid.

## Introduction

Lung fibrosis is a common trait of different diseases with different etiologies and different pathophysiological pathways. Lung fibrotic disorders are characterized by accumulation of fibroblasts, myofibroblasts and extra-cellular matrix leading to chronic respiratory failure. The origin of the fibroblasts remains debated and is probably multiple, the respective role of the proliferation of resident fibroblasts or mesenchymal precursors, of the transition of epithelial, endothelial or mesothelial cells toward a mesenchymal phenotype, or the recruitment to the lung of circulating blood mesenchymal precursors is debated [1], [2].

In experimental models of acute lung injury (ALI), bone-marrow derived cells have been shown to be recruited to the lung and to contribute to normal and pathologic repair [3]. Among these cells, fibrocytes are mesenchymal progenitors derived from hematopoïetic precursors, which co-express leukocyte (CD45+) and fibroblast markers (collagen1+) [4]. Fibrocytes are implicated in a wide variety of focal and diffuse remodeling disorders [5], in the skin, lung, liver, kidney, pancreas or in atherosclerosis [6]. The fibrocytes, and their CD14 positive mononuclear precursors [6], are recruited from the blood to organs through multiple chemokine signaling pathways particularly including the CCL2/CCR2 and the CXCL12/CXCR4 pathways [6]. In murine models of ALI, the inhibition of fibrocyte recruitment [7] or the blocking of their differentiation from precursors [8] has been associated with a dramatic limitation of lung fibrosis development. In the blood, fibrocytes could represent almost 1% of the nucleated cells in healthy subjects, 2.7% in stable idiopathic pulmonary fibrosis (IPF) and 14.5% in acute IPF exacerbation (AE) [2]. In patients with IPF, an increased number of circulating fibrocytes has been associated with poor outcome [2]. Fibrocytes have been implicated in the pathophysiology of other fibrotic lung disorders, such as interstitial lung disease associated with systemic sclerosis (SSc-ILD), where fibrocytes have been shown to be increased in the peripheral blood and to accumulate in the lung [9]. In patients with acute lung injury (ALI), we described that fibrocytes could be detected in bronchoalveolar lavage (BAL) in almost all patients and that BAL fibrocyte percentage was independently associated with increased mortality [10].

We asked whether fibrocytes could be recruited to the alveolar space in IPF and SSc-ILD and if the detection of fibrocytes in BAL would indicate a worse prognosis in those patients. Furthermore, we determined wether fibrocytes detection was associated with the capacity to grow fibroblasts from BAL.

## Patients and Methods

### Ethics Statement

The study was approved by the Institutional Review Board of the French learned society for respiratory medicine -Société de Pneumologie de Langue Française. Oral informed consent was obtained from the participants to the study at the time of bronchoscopy and recorded in the clinical charts. Clinical and functional data were collected on a standardized and anonymous collection form.

### Methods

A BAL was performed for a diagnostic purpose when a diagnosis of IPF or SSc-ILD was suspected from March 2006 to August 2008, with standard procedures [11]. When enough material was available, BAL was used for research purpose and processed as previously described [11]. Patients fulfilled the 2000 ATS/ERS criteria for diagnosis of IPF (with surgical lung biopsy in 6 patients), and retrospectivelly fullfilled the 2011 criteria [12], [13]. SSc was defined according to international criteria [14]; an ILD was based on the chest CT-scan. The control group included patients without CT-scan evidence of ILD who required a bronchoscopy for the diagnosis of unexplained cough (n = 4) or lung nodule (n = 3), suspicion of haemoptysis (n = 2), staging of pharyngolaryngeal cancer (n = 2). Nine of them were smokers. In all cases bronchoscopy did not display any abnormality.

Pulmonary hypertension was defined by PAPs>40 mm Hg assessed by echocardiography. CT-scan scoring of ground-glass opacities and fibrosis was made according to Kazerooni score [15]. AE of IPF was defined according to the Collard criteria [16]. Progression of fibrosis was defined by a decrease of vital capacity of more than 10% or a decrease of DLCO of more than 15% after exclusion of an AE [17].

#### BAL processing and flow cytometry analysis

The BAL was performed and processed as previously described [11]. A differential BAL cell count was performed on a cytocentrifuge smear with a Diff quik stain kit (Dade International, Miami, USA). The viability of the cells was determined by Trypan blue exclusion, and always exceeded 95%.

Fibrocytes (CD45+, Collagen1+) were analyzed by flow cytometry analysis as described elsewhere [10]. A part of BAL cell pellet obtained after centrifugation was stored in Cyto-chex storage solution (Streck, La Vista, NE) (1 × 106 cells/mL) for <1 wk at 4°C before analysis. The cells were resuspended in 50 µl of staining buffer (0.2% BSA and 0.02% sodium azide in PBS) and 1 µl of Fc blocker (BD Biosciences) for 10 min, then washed three times with staining buffer and permeabilized by adding BD Cytofix/Cytoperm kit (BD-Pharmingen, San Diego, CA) and incubated with monoclonal mouse antihuman collagen-1 antibody (Chemicon International, Temecula, CA) or IgG1 isotype control for 30 mins. Cells were washed twice, prior labeling with goat antimouse antibody conjugated to fluorescein isothiocyanate (Nordic immunologic Laboratories, Tilburg, The Netherlands). Next, the cells were stained for surface antigen CD45 (CD45-APC; BD Pharmingen) for 15 mins. Cells were washed twice and immediately analyzed.

Flow Cytometry was performed utilizing FACScalibur flow cytometer (BD-Biosciences). For each sample at least 10,000 events were collected. Data were analyzed using FlowJo® software (Tree Star, Inc. Ashland, USA). The negative threshold for all markers was set using the matched IgG isotype control. Fibrocytes numbers were expressed as absolute number per milliliter of BAL fluid.

#### Fibroblast culture from BAL

To grow fibroblasts, the BAL cells were cultured for at least 28 days and characterized as previously described [18]. Briefly the BAL cell viability was determined before initial plating by Trypan blue vital staining. Cells grown from BAL on Lab-Tek slides (Nunc, Naperville, IL, USA) were used for immunocytochemical characterisation 24 h and 21 days after initial plating and then at passage 1 and 3.

#### Immunocytochemistry

The alveolar cells were fixed with acetone. Anti-human antibodies (Ab) directed against collagen 1 (Sigma Aldrich, St Louis, MO, USA), vimentin (Immunotech, Marseille, France), prolyl-4-hydroxylase (α-4H), desmin, pancytokeratin, CD31, α-SMA, CD45 (Dako SA, Trappes, France), smooth muscle myosin 1 (Abcam, Paris, France), CD14, CD34 (BD Pharmingen, Le Pont-De-Claix, France) or isotype-matched control Ab were used. Positive cells were revealed using the Vectastain ABC-alkaline phosphatase kit mouse immunoglobulin G (Vector Abcys, Paris, France) and the fast red substrate (Dako SA).

#### Confocal microscopy analysis

Cells cultured on Lab-Tek slides were fixed after 21 days of culture with acetone and saturated with 50 mM NH4Cl and permeabilised with 0.1% Triton X100. After saturation with normal horse serum, slides were incubated with anti-collagen 1 monoclonal Abs (1∶290) (Sigma Aldrich) prior to the addition of fluorescent-labelled Abs (biotinilated horse antimouse (1∶300) plus Alexa Fluor 568-conjugated streptavidin (1∶100); Invitrogen, Carlsbad, CA, USA). After washing, the cells were incubated with anti-CD45-Alexa Fluor 488 Abs (1∶25) (Santa Cruz Biotechnology, San Diego, CA, USA). Cell nuclei were localised by DNA staining with thiazole orange protein 3 (0.4 µg·mL−1; Molecular Probes, Eugene, OR, USA). Slides were mounted with Prolong Gold anti-fade reagent (Invitrogen) and examined by confocal laser scanning microscopy (LSM-510-META microscope; Zeiss, Oberkochen, Germany). To test for the specificity of immunostaining, Abs were replaced by an isotype-matched control Ab.

#### Chemokines measurements in BAL

CCL2 and CXCL12 concentrations were measured in BAL fluid supernatant by ELISA (Quantikine and Duoset Kits, R&D systems, Abingdon,UK). The detection threshold of the assays were 15 and 16 pg/ml for CCL2 and CXCL12 respectively. When a chemokine concentration was below the detection level of the assay, it was attributed a value of 0.

### Statistical Analysis

As the distribution of the % of fibrocytes in the population studied was not normal, and as the number of patients studied in every group was below 30, non parametric tests were used for statistical analysis. Continuous variables were expressed as the median (range) and compared by the Mann-Whitney U test. Categorical variables were expressed as counts and proportions and compared by the Fisher’s exact test. Correlations were assessed with the Spearman correlation test Survival was assessed using the Kaplan–Meier estimator. All tests were two-sided, with p<0.05 indicating statistical significance. All the analyses involved use of the software GraphPad Prism.

## Results

### Patient Characteristics

Twenty-six patients with IPF, 9 patients with SSc-ILD (cutaneous involvement was diffuse in 8 patients and limited in one) and 11 control patients were included (Table 1 and 2). The HRCT pattern was highly suggestive of nonspecific interstitial pneumonia in 8/9 SSc patients, and suggestive of usual interstitial pneumonia in one SSc patient only. Mean follow-up was 22 months [0–53]. At the end of follow-up, 10 IPF and one SSc patients were deceased, one SSc patient had a lung transplant, 5 patients with IPF and 2 patients with SSc presented a slow progression of fibrosis. The other patients were stable.

**Table 1:** Clinical and functional characteristics of the patients.

	IPFN = 26	SSc-ILDN = 9	ControlsN = 11
Age *(years)*	74 [56–84]	62 [46–72]*	68 [34–79]*
Gender (Male)	19 (73%)	4 (44%)	9 (81%)
Smoker (ever)	16 (61%)	2 (22%)	9 (81%)
Delay since diagnosis*(days)*	17.5 [0–655]	146 [5–525]	na
Oral Steroids	6 (23%)	1 (11%)	0
Dose (mg)	8.7 [5–40]	10	na
Exacerbation	3 (11%)	0	na
Progression	13 (48%)	0	na
Lung Function Tests			
TLC *(% pred)*	68 [28–105]	56 [35–94]	88 [50–118]*
VC *(% pred)*	75 [34–108]	57 [29–134]	102 [52–109]
DLCO *(% pred)*	35 [15–91]	33 [16–85]	Not available
PaO2 *(mmHg)*	76 [45–101]	90 [39–99]	Not available
Pulmonaryhypertension (N)	3 (11%)	3 (33%)	0

Data are expressed as median [range] or N (%); na: not applicable. *p<0.05.

**Table 2:** Bronchoalveolar lavage results.

	IPFN = 26	SSC-ILDN = 9	ControlsN = 11
Cellularity (x10^3^/ml)	150 [72–1000]	310 [48–450]	180 [30–750]
Macrophages (%)	80 [37–95]	88 [63–92]	86 [70–94]
Lymphocytes (%)	6 [0–20]	9 [2]–[26]	10 [1]–[22]
Neutrophils (%)	10 [2–90]	6 [0–10]	4 [1]–[17]
Eosinophils (%)	2 [0–17]	1 [0–10]	0 [0–6]
Fibrocytes detection	14 (53%)	5 (55%)	0 (0%)*
Fibrocytes/ml	5240 [370–29500]	9700 [4815–13650]	0*
CCL2 (pg/ml)	137 [0–1033]	212 [63–315]	37 [14–123]
CXCL12 (pg/ml)	0 [0–23]	0 [0–22]	0 [0–0]
Fibroblast culture (+)	12 (46%)	5 (55%)	0 (0%)

Data are expressed as median [range] or N (%). *p<0.05.

### Detection of Fibrocytes in BAL

**Figure 1:**
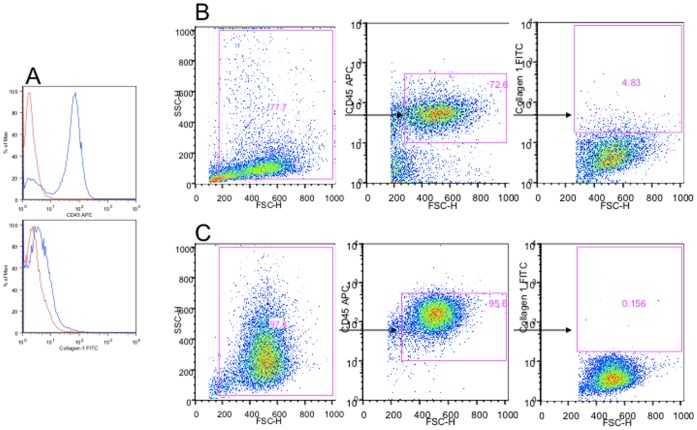
Representative flow cytometric analysis of bronchoalveolar lavage cells from a patient with idiopathic pulmonary fibrosis (IPF) and from a control patient. Flow cytometry was used to determine the percentage of fibrocytes. **A)** Cells were stained for CD45 and collagen 1, and analyzed by flow cytometry. Isotype control staining of cells is shown as red histograms and CD45 or collagen 1 as blue histograms. The negative threshold for all markers was set using the matched IgG isotype control. **B)** Sample analysis from one patient with IPF: In the left panel, forward scatter/side scatter (FSC/SSC) dot-plot, unstained cells were gated to exclude debris. In the middle panel, CD45+ cells were gated. In the right panel, CD45+ collagen 1+ cells were gated. **C)** Sample analysis from one control patient. In the left panel, forward scatter/side scatter (FSC/SSC) dot-plot, unstained cells were gated to exclude dead cells. In the middle panel, CD45+ cells were gated. In the right panel,CD45+ collagen 1+ cells were gated. The shape of the gate was chosen to account for autofluorescence. Fluorescein isothiocyanate, FITC; allophycocyanin, APC; forward scatter, FSC-H; side scatter, SSC-H.

**Figure 2:**
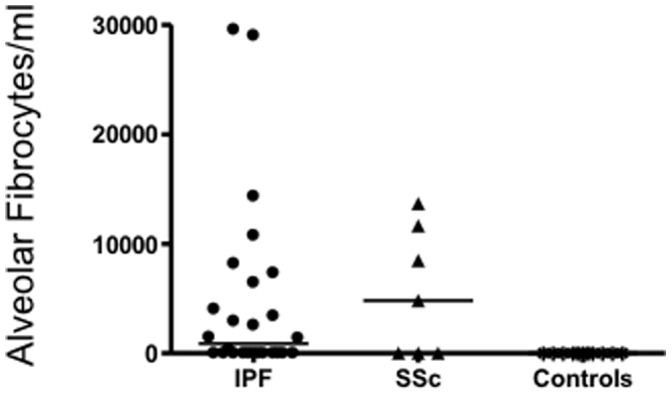
Number of fibrocytes in bronchoalveolar lavage in idiopathic pulmonary fibrosis (IPF), Scleroderma patients with interstitial lung disease (SSc) and in controls. Fibrocytes were never detected in controls and were in median 5240/ml and 9700/ml in IPF and SSc patients respectively.

Alveolar fibrocytes, defined as cells expressing CD45 and collagen 1, were detected in 14/26 patients in the IPF group (53%), in 5/9 patients in the SSc-ILD group (55%) and in 0/11 controls (p = 0.006 and p = 0.02 versus controls, respectively). Figure 1 shows a typical image of FACS analysis for one IPF patient and one control. Alveolar fibrocytes were in median 5240/ml [370–29500] and 9700/ml [4815–13650] representing 0.67% [0.4–19.7] and 2.73% [0–3.9] of the BAL cells, in IPF and SSc-ILD patients respectively (Figure 2). In 3 patients, the BAL was performed at the time of an acute exacerbation of IPF. In those patients, fibrocytes were detected in one BAL (8177 fibrocytes/mm^3^) and were absent in two BAL. In view of the limited number of SSc BAL evaluated, further analyses were limited to IPF patients.

In IPF patients, the number of fibrocytes in BAL positively correlated with BAL macrophage counts (p = 0.0006, r = 0.62), and negatively with the BAL neutrophil counts (p = 0.0016, r = 0.58), but did not correlate with lymphocyte or eosinophil counts. We identified no link with infection as no pathogen grew from these BAL fluids at significant concentrations.

**Figure 3:**
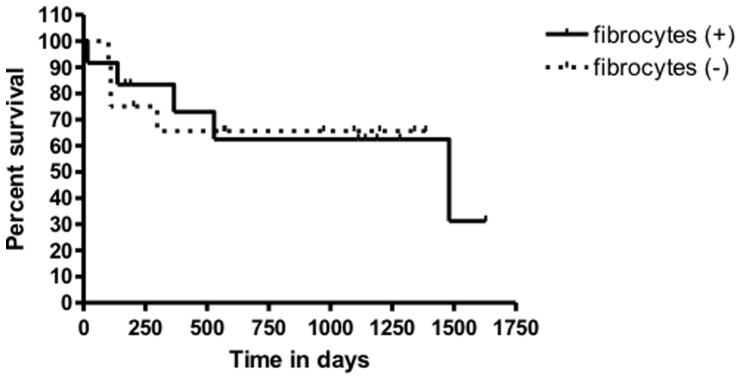
Kaplan-Meier curve of time to death in IPF patients with (solid line) or without (dotted line) alveolar fibrocytes. Survival did not differ between the 2 groups.

IPF patients with detection of fibrocytes in BAL did not differ from patients without fibrocytes in any of the clinical, biological or radiological criteria evaluated (Figure 3, table 3). In IPF patients, the number of BAL fibrocytes did not correlate with age, tobacco use, time since diagnosis, quantitative quotation of ground glass opacities, lung volumes or outcome assessed by the risk of progression, AE, tranplantation or death. However, an increased number of BAL fibrocytes was associated with higher DLCO (p = 0.01, r = 0.5), higher arterial blood PaO2 at rest (p = 0.08, r = 0.33) and a lower lung CT-scan score of fibrosis (p = 0.04, r = 0.4). The number of alveolar fibrocytes did not correlate with the alveolar concentration of CCL2 and CXCL12 (table 2).

**Table 3:** Clinical and functional characteristics of the IPF patients according to the detection of fibrocytes in BAL.

	Fibrocytes(+)	Fibrocytes(−)	P value
Number of patients (%)	14 (54%)	12 (46%)	
Age	73 [56–83]	75 [65–84]	0.49
Gender (Male)	12 (85%)	7 (58%)	0.12
Smoker (ever)	9 (64%)	7 (58%)	1
Exacerbation	1 (7%)	2 (28%)	0.58
Delay since diagnosis (days)	13 [0–655]	17 [0–250]	0.45
Oral steroids	3 (21%)	3 (25%)	1
Death or lung transplant	6	4	0.7
Pulmonary Hypertension (N)	0	3	0.08
HRCT fibrosis score	8 [4]–[14]	8 [6]–[13]	0.98
Fibrocyte count/ml	5240 [369–29550]	0 [0–0]	
CCL2 (pg/ml)	106 [0–350]	182 [11–1033]	0.14
CXCL12 (pg/ml)	0 [0–0]	0 [0–22.7]	0.37
*Lung function tests*			
VC *(% pred)*	79 [34–108]	70 [40–96]	0.7
DLCO *(% pred)*	45 [15–91]	45 [58–73]	0.06
PaO2 *(mm Hg)*	78 [45–101]	76 [59–92]	0.48

Data are expressed as median [range] or N (%).

#### Evolution of BAL fibrocytes counts over time

**Figure 4:**
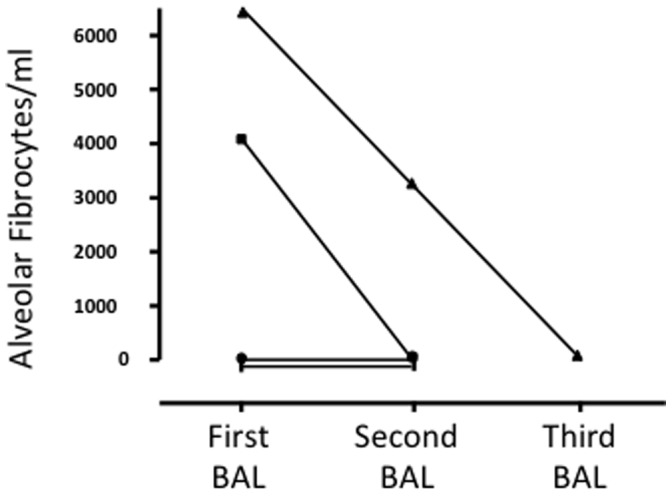
Evolution of the number of alveolar fibrocytes in 4 patients with IPF where bronchoalveolar lavage fluid was obtained at two or more occasions. Alveolar fibrocytes were no more detectable in the 2 patients with initial detectable alveolar fibrocytes. In the 2 patients without detectable alveolar fibrocytes at initial evaluation, fibrocytes remained undetectable in subsequent broncholveolar lavage fluid analysis.

We had the opportunity to study the evolution of BAL fibrocytes count in three IPF patients who required a second BAL procedure (respectively 8, 95 and 337 days after the first BAL), and in one IPF patient who required two BAL procedures (respectively 260 and 642 days after the first BAL). In all cases, the BAL were performed for a clinical purpose. Alveolar fibrocytes were detectable in the first BAL in 2/4 patients. The alveolar fibrocytes were no more detectable in the second lavage for one of them, diminished in the second and were no more detectable in the third lavage (Figure 4).

#### Fibroblast culture from BAL

**Figure 5:**
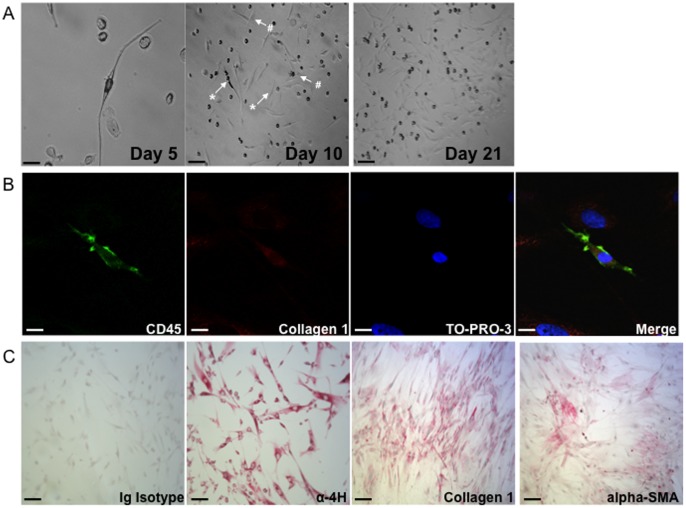
Characterization of bronchoalveolar lavage cells in culture. A) Morphology of adherent bronchoalveolar lavage (BAL) cells at amplification when a positive culture occurred. From left to right: after 5 days (scale bar  =  25 µm), at 10 days (scale bar  =  250 µm) and at 21 days (scale bar  =  250 µm). The arrows indicate typical adherent mesenchymal cells with high (*, fibrocyte phenotype) or low ratio (#, fibroblast phenotype) of cell length to cell width. **B)** Confocal microscopy analysis of fibrocytes (spindle-shaped cells co-expressing CD45 and collagen) in BAL cell culture at day 21. From left to right: CD45, collagen 1, thiazole orange protein 3 (TO-PRO-3) and merge of the three fluorescence channels. Scale bars  =  15 µm. **C)** Representative immunocytochemical stain prepared from alveolar fibroblasts at passage 1. From left to right: Ig Isotype, α-propyl-4-hydroxylase (α-4H), collagen 1, α-smooth muscle actin (a-SMA). Scale bars  =  100 µm.

The morphological and immunohistochemical characteristics of cells grown from BAL are shown in Figure 5. The cells were spindle shaped and expressed collagen 1, vimentin and prolyl-4-hydroxylase, without expression of desmin, pancytokeratin, CD14, CD31, or CD34.After 28 days of culture, fibroblasts grew from 0/9 of the BAL control cultures (two cultures were stopped because of infection), 12/26 BAL of the IPF patients (P = 0.014 versus controls) and 5/9 of the SSc-ILD patients (P = 0.03 versus controls). Fibroblasts grew from 2/3 BAL performed in patients with AE.

The IPF patients were divided according to the cell culture results. The clinical, functional and radiographical parameters and survival were similar in the two groups (table 4). BAL cytology was similar in the two groups. Fibroblast culture positivity rate was similar in BAL where we detected fibrocytes initially (5/14) as compared with BAL where fibrocytes were not detected (7/12, P = 0.43). The positivity of fibroblast culture was associated with an increased concentration of CCL2 in BAL fluid at 262 pg/ml [102–1033] vs 64 pg/ml [0–341] in negative cultures, but not with CXCL12.

**Table 4:** Clinical and functional characteristics of the IPF patients with positive or negative cultures of fibroblasts.

	Fibroblasts (+)	Fibroblasts (−)	P value
Number of patients (%)	12 (46%)	14 (54%)	
Age	75 [66–84]	72 [56–84]	0.4
Gender (Male)	7 (58)	12 (72)	0.06
Smoker (ever)	8 (66)	8 (57)	0.7
Exacerbation	2	1	0.58
Delay since diagnosis (days)	18 [0–236]	7 [0–655]	0.74
Death or lung transplant during follow up	4 (33)	4 (42)	1
Oral steroids	4 (33)	2 (14)	0.36
CCL2 (pg/ml)	269 [102–1033]	64 [0–341]	0.02
CXCL12 (pg/ml)	0 [0–22.7]	0 [0–0]	0.54
PaO2 *(mm Hg)*	76 [50–101]	77 [45–91]	0.8
VC *(% pred)*	79 [40–101]	67 [34–108]	0.57
DLCO *(% pred)*	35 [15–59]	35 [27–91]	0.36
Pulmonary Hypertension	1	2	1
HRCT fibrosis score	8 [4]–[13]	8 [6]–[14]	0.56
Detection of Fibrocytes in BAL	5 (41)	9 (64)	0.43

Data are expressed as median [range] or N (%).

## Discussion

This study demonstrates that fibrocytes are detected in BAL in almost 50% of IPF or SSc-ILD patients whereas these cells are always absent from control BAL, and that in IPF patients, the number of alveolar fibrocytes correlates with the number of alveolar macrophages, and with markers of less severe disease : DLCO, CT-scan score of fibrosis and PaO2. However, our results do not support the presence or absence of BAL fibrocytes as a prognostic marker in those patients.

This is the first study to identify fibrocytes in the alveolar milieu in patients with fibrotic lung disorders. Fibrocytes are monocyte-derived cells that have features of macrophages and fibroblasts [19]. Increased numbers of circulating fibrocytes have been previously reported in patients with IPF or SSc [2], [20] [21] and in the lung of IPF and SSc patients [22] [9]. Andersson-Sjoland and colleagues showed that fibrocytes could be detected in 8/9 IPF lung samples studied and that the number of fibrocytes in lung tissue correlated with the number of fibroblastic foci, suggesting that the number of fibrocytes was an index of the ongoing local fibrotic process [22]. The detection of fibrocytes in BAL fluid could be a minimally invasive way to assess the number of fibrocytes in lung tissue and the severity of fibrogenic process. However, we detected fibrocytes in BAL only in 50% of the patients studied and the presence of fibrocytes in BAL fluid did not associate with the burden of fibrotic tissue as assessed by CT-scan nor with the severity of the disease as assessed by lung function tests results and blood gas abnormalities. In fact we observed the opposite, since higher numbers of fibrocytes were associated with better FVC, better DLCO and lower fibrotic score on HRCT in the patients where fibrocytes could be detected. This observation is surprising as most of the literature available associates circulating fibrocytes numbers with worse prognosis particularly in humans [2], [7], [8], [10], [23]. Interestingly, Moeller and colleagues observed that the number of circulating fibrocytes did associate with a reduced survival in a population of IPF patients, whereas they found no correlation between circulating fibrocyte counts and lung function parameters (FVC, TLC, DLCO), 6 minute walking distance or HRCT scores [2]. Our interpretation is that fibrocytes count and differentiation depends on the compartment studied (blood, tissue or BAL for instance) as the differentiation of monocytes to fibrocytes and from fibrocytes to fibroblasts depends greatly on the local microenvironment [24].

The origin of BAL fibrocytes is not unequivocal. Circulating fibrocytes could directly be attracted to the alveolar space because of the local expression of chemokines for fibrocytes (such as CCL2 or CXCL12) already demonstrated in lung fibrosis [22], [25]. It is worthnoting that Andersson-Sjoland and colleagues measured CXCL12 levels in BAL from 20 IPF patients and 5 controls using the Quantikine assay (R&D systems). They found that CXCL12 was never detected in BAL from controls whereas it was detected in 8 out of 20 (40%) IPF BAL samples. In our hands, CXCL12 was detected only in 1/14 samples analyzed using an assay of similar sensitivity (Quantikine and Duoset Kits, R&D systems). We do not have a clear explanation for this discrepancy. Degradation of the cytokine during the BAL storage is unlikely as we systematically added aprotinin, a broad spectrum serine protease inhibtor, to BAL supernatants.

It is also probable that blood monocytes penetrating the alveolar space in patients with ongoing lung fibrosis could differentiate into fibrocytes, since profibrotic mediators such as IL-4, IL-13, TGF-β and endothelin-1 are present in the fibrotic alveolar milieu and have been shown to induce the differentiation of monocytes to fibrocytes in vitro [19]. According to their origin, it is highly probable that the differentiation markers expressed by fibrocytes could differ. Furthermore, in vitro experiments have shown that fibrocytes may change their differentiation markers with time [26].

We observed that the number of alveolar fibrocytes was positively correlated with the number of alveolar macrophages and negatively correlated with the number of alveolar neutrophils. As most fibrocytes arise from the differentiation of blood monocytes in tissue, the correlation with monocyte/macrophage count was not unexpected, as we observed the same correlation in patients with ALI/ARDS [10]. The negative correlation between the number of alveolar fibrocytes and the number of alveolar neutrophils could simply reflect the fact that the more macrophages you have, the less neutrophils you have. However, neutrophils could also modulate monocyte to fibrocyte differentiation or fibrocyte recruitment. This has not been specifically studied before but it is scientifically plausible as neutrophil elastase has been shown to cleave both CXCL12 and its receptor CXCR4 [27] and to deactivate endothelial bound CXCL12 and inhibit the subsequent T lymphocytes transendothelial migration [28].

We observed that the number of alveolar fibrocytes inversely correlated with the severity of lung disease as assessed by DLCO, score of fibrosis and PaO2. Most importantly, fibrocytes counts were not increased in patients with AE of IPF, and when BAL were repeated we observed a decrease of the number of alveolar fibrocytes. Altogether, these observations suggest that alveolar fibrocytes expressing CD45 and collagen-1 could be a marker of early fibrotic disease, and that the differentiation of fibrocytes evolve with time.

Fibrocytes differentiate into fibroblast in vitro when appropriately stimulated [19]. There is a debate as to whether fibrocytes can differentiate toward a myofibroblastic phenotype. In this study, we observed that the capacity to grow fibroblasts from BAL did not correlate with the number of alveolar fibrocytes. Other fibroblast precursor are present in BAL, such as local mesenchymal stem cells. Lama and colleagues showed that BAL fibroblast which grew from lung transplant patients were of donor origin, thus demonstrating the role of the local mesenchymal cell precursors in bronchiolitis obliterans due to lung transplant [29]. Other investigators have already shown that fibroblast could be cultured from BAL of IPF and SSc-ILD patients, with negative cultures from control BAL [30]. Surprisingly, although fibroblast could be cultured from almost 50% of the BAL from IPF or SSc-ILD patients and never from control patients, we did not evidence any difference between groups with or without positive cultures.

Our study has some limitations. As we did not collect blood at the time of BAL, we were not able to measure circulating fibrocytes although it would be interesting to compare circulating and alveolar fibrocytes in the same patient. The second limit is the relatively low number of patients included.

In summary, we were able to detect fibrocytes in BAL fluid in about half of the patients with IPF and SSc-ILD. Fibrocytes detection was not associated witht the capacity to grow fibroblast from BAL fluid. Further studies are needed to investigate the role of alveolar fibrocytes and their interaction with circulating fibrocytes.
